# Preface

**DOI:** 10.1007/s11047-016-9568-z

**Published:** 2016-07-22

**Authors:** Marian Gheorghe, Gheorghe Păun, Mario J. Pérez-Jiménez, Agustín Riscos-Núñez

**Affiliations:** 1 Bradford, UK; 2 Bucharest, Romania; 3 Sevilla, Spain

Membrane computing is an area of natural computing, initiated in 1998, which studies models of computation inspired by the structure and functioning of living cells, and the organization of cells in tissues and other structures, including the brain. The resulting models (called *P systems*) are distributed parallel computing devices, processing multisets in compartments defined by membranes.

Many classes of P systems were considered, with motivations coming from biology, computer science, mathematics, and applications. Most of them are computationally universal and, if an exponential working space can be produced in polynomial time (e.g., by membrane division) during the computation, then they are able to solve computationally hard (typically, **NP**-complete) problems in a feasible (typically, polynomial) time. A series of applications were reported, especially in biology and bio-medicine, but also in computer graphics, cryptography, linguistics, economics, approximate optimization, and robot control. Several simulation programs (useful in applications), including a specialized programming language, *P-lingua*, are available by now.

A comprehensive information about this research area (considered in 2003 by ISI as “fast emerging research front in computer science”) can be found at the Website http://ppage.psystems.eu, while a systematic presentation (at the level of year 2009) can be found in *The Oxford Handbook of Membrane Computing*, edited by Gh. Păun, G. Rozenberg, A. Salomaa, Oxford Univ. Press, 2010.

Three international meetings dedicated to membrane computing take place each year: the Conference on Membrane Computing, CMC (the 16th edition in 2015 was held in Valencia, Spain), the Asian Conference on Membrane Computing, ACMC (the fourth edition was held in 2015 in Heifei, China), and the Brainstorming Week on Membrane Computing, BWMC. Excepting the first edition (2003), all subsequent editions of the Brainstorming Week were organized by the Research Group on Natural Computing (RGNC) of the Department of Computer Science and Artificial Intelligence of the Universidad de Sevilla, Spain.

The 13th edition of the Brainstorming (Sevilla, from February 2 to February 6, 2015) had a special focus on the interplay between membrane computing and high performance computing.

In the style of previous meetings, the 13th edition of BWMC was conceived as a period of active interaction among the participants, with the emphasis on exchanging ideas and cooperation. Several “provocative” talks were delivered, mainly devoted to open problems, research topics, and conjectures, followed by an intense cooperation among the 30 participants.

The efficiency of this type of meetings was again proved to be very high and a volume containing working papers elaborated during the meeting and completed shortly after that was published by Fenix Editora, Sevilla, 2015 (it can be found at the membrane computing website mentioned above).

Six papers from this volume, together with three papers presented at ACMC 2015 (November 12–15, 2015, Anhui University, Hefei, Anhui, China), were selected for this special issue of *Natural Computing*. Similar special issues of international journals were published after all Brainstorming Week meetings organized in Sevilla.

The papers were thoroughly reworked after the meeting and then they went through the standard refereeing procedure of the journal.

Besides the quality criterion, the selection was also concerned with the balance between the addressed topics, so that this special issue covers a variety of subjects currently investigated in membrane computing. All these papers contain not only sound new results, but also stimulating ideas and research topics for further investigations. They cover a broad range of topics, from theoretical aspects such as computational power and complexity, and relationships with other computational models, to efficient implementations of P systems and evolutionary approaches solving various optimization problems. The paper “Simulating P systems with membrane dissolution in a chemical calculus”, by B. Aman, P. Battyányi, G. Ciobanu and Gy. Vaszil, presents a mapping of some classes of P systems into formulas of the chemical calculus. F. G. C. Cabarle, H. N. Adorna and M. J. Pérez-Jiménez in “Notes on spiking neural P systems and finite automata” investigate the potential of spiking neural P systems to simulate the behaviour of deterministic finite automata and state transducers. “A class of restricted P colonies with string environment”, by L. Cienciala, L. Ciencialová and E. Csuhaj-Varjú, studies the computational power of a class of P colonies using an environment specified as a string. “Monodirectional P systems”, by A. Leporati, L. Manzoni, G. Mauri, A. E. Porreca and C. Zandron, investigates the computational complexity of P systems where communication happens from a membrane towards its parent. “Parallel simulation of population dynamics P systems: updates and roadmap”, by M. A. Martínez-del-Amor, L. F. Macías-Ramos, L. Valencia-Cabrera and M. J. Pérez-Jiménez, reports further work on simulating population stochastic P systems on parallel hardware platforms such as GPUs. L. Pan and Gh. Păun in “On the universality of purely catalytic P systems” prove that purely catalytic P systems with one catalyst are universal when promoters and inhibitors or promoters and a priority relationship are used, and purely catalytic cooperating P systems with two catalysts are universal. J. Xiao, J. He, P. Chen and Y. Niu in “An improved dynamic membrane evolutionary algorithm for constrained engineering design problems” show the efficiency of combining the dynamic membrane structure of P systems with the particle swarm optimization and differential evolution by applying the method to a set of benchmark problems. “P systems based computing polynomials: design and formal verification”, by W. Yuan, G. Zhang, M. J. Pérez-Jiménez, T. Wang and Z. Huang, presents some deterministic transition P system solutions to the problem of computing the value of a polynomial; the validity of the method is proved and its descriptive complexity assessed. X. Zhang, J. Li and L. Zhang in “A multi-objective membrane algorithm guided by the skin membrane” propose a multi-objective membrane algorithm and show for a number of benchmark problems that it outperforms state-of-the-art multi-objective optimization algorithms.

As mentioned above, the Brainstorming Week was organized by the Research Group on Natural Computing from Universidad de Sevilla,^1^ and all the members of this group were enthusiastically involved in this work. The ACMC event was organized by the Anhui University.^2^ Thanks to the organizers for help and support. Thanks also to the authors and the referees, for their highly professional and timely work.

The Brainstorming Week meeting was supported from various sources: (1) Proyecto del Ministerio de Economía y Competitividad, grant TIN2012 37434 (cofinanced by FEDER funds), (2) Fundación para la Investigación y el Desarrollo de las Tecnologías de la Información en Andalucía (FIDETIA), (3) Instituto de Matemáticas de la Universidad de Sevilla (IMUS), (4) V Plan Propio de Investigación de la Universidad de Sevilla, as well as by the Department of Computer Science and Artificial Intelligence from Universidad de Sevilla.

The editors

January 2016

## Footnotes

1 Website http://www.gcn.us.es.

2 Website http://gjc.ahu.edu.cn/en/.

